# Enhanced transduction of colonic cell lines *in vitro *and the inflamed colon in mice by viral vectors, derived from adeno-associated virus serotype 2, using virus-microbead conjugates bearing lectin

**DOI:** 10.1186/1472-6750-7-83

**Published:** 2007-11-28

**Authors:** Samuel J Farlow, Alan Jerusalmi, Takeshi Sano

**Affiliations:** 1 Center for Molecular Imaging Diagnosis and Therapy and Basic Science Laboratory, Department of Radiology, Beth Israel Deaconess Medical Center, Harvard Medical School, Boston, Massachusetts 02215, USA; 2 Current Address : AVEO Pharmaceuticals, Inc., Cambridge, Massachusetts 02139, USA

## Abstract

**Background:**

Virus-mediated delivery of therapeutic transgenes to the inflamed colon holds a great potential to serve as an effective therapeutic strategy for inflammatory bowel disease, since local, long-term expression of the encoded therapeutic proteins in the colorectal system is potentially achievable. Viral vectors, derived from adeno-associated virus (AAV), should be very useful for such therapeutic strategies, particularly because they can establish long-term expression of transgenes. However, few studies have been carried out to investigate the ability of AAV-based vectors to transduce the inflamed colon.

**Results:**

AAV, derived from adeno-associated virus serotype 2 (AAV2), showed a limited ability to transduce colonic cell lines *in vitro *when used in free form. No appreciable enhancement of the transduction efficiency was seen when AAV2 particles were attached stably to the surfaces of microbeads and delivered to target cells in the form of AAV2-microbead conjugates. However, the transduction efficiency of these colonic cell lines was enhanced substantially when a lectin, concanavalin A (Con A), was co-attached to the microbead surfaces, to which AAV2 particles had been conjugated. This considerable infectivity enhancement of AAV2-microbead conjugates by the co-attachment of Con A may be derived from the fact that Con A binds to α-D-mannosyl moieties that are commonly and abundantly present in cell-surface carbohydrate chains, allowing the conjugates to associate stably with target cells. Intracolonical administration of free AAV2 or AAV2-microbead conjugates without Con A into a mouse colitis model by enema showed very poor transduction of the colonic tissue. In contrast, the delivery of AAV2 in the form of AAV2-microbead conjugates bearing Con A resulted in efficient transduction of the inflamed colon.

**Conclusion:**

AAV2-microbead conjugates bearing Con A can serve as efficient gene transfer agents both for poorly permissive colonic cell lines *in vitro *and for the inflamed colon in a mouse colitis model. This efficient transduction system for the inflamed colon should be useful for the development of gene therapy strategies for inflammatory bowel disease.

## Background

Inflammatory bowel disease (IBD), consisting of two idiopathic inflammatory disorders, Crohn's disease and ulcerative colitis, is characterized by chronic intestinal inflammation, which causes severe destruction of the mucosa of the colorectal system [1-4]. An intense, local immune response is like to be responsible for the initiation and progress of the inflammatory process. A variety of pro-inflammatory cytokines, such as tumor necrosis factor-α (TNF-α), interleukin-1β (IL-1β), IL-2, IL-12, and interferon-γ, are involved in these inflammatory processes. Thus, one potential therapeutic strategy for IBD is to repress these inflammatory processes by using anti-inflammatory cytokines, extracellular domains of the receptors of pro-inflammatory cytokines, and antagonists and antibodies against pro-inflammatory cytokines. Gene therapy approaches are particularly attractive for such therapeutic strategies, since local, long-term expression of therapeutic proteins in the colorectal system is potentially achievable. Previous studies primarily used adenoviral vectors (Ad5), derived from adenovirus serotype 5, as gene transfer agents for the inflamed colon. Although the colorectal system is readily accessible externally, the presence of the mucous coat on the colonic epithelium and the dynamic fluidic properties of the colorectal system act as barriers for the access to the colonic tissue by viral vectors that are administered intracolonically. Thus, administration of relatively large amounts of Ad5 was required to achieve sufficient levels of transgene expression in the colon [5-7]. We recently showed by using Ad5 with a mouse chemically induced colitis model that the transduction efficiency of the inflamed colon by Ad5 was very poor when administered intracolonically in free form, but that it was enhanced considerably when Ad5 was used in the form of Ad5-microbead conjugates bearing a lectin, concanavalin A (Con A), which serves as an effective anchoring agent for the conjugates [8]. When an Ad5 construct carrying the gene for mouse IL-10, a potent anti-inflammatory cytokine that is among the most promising protein therapeutics for IBD [9-19], was administered intracolonically in the form of Ad5-microbead conjugates bearing Con A, a considerable increase in the local IL-10 level in the colon was seen. This suggests that intracolonical administration of viral vectors carrying the genes for IL-10 and other therapeutic proteins may serve as an effective therapeutic strategy for IBD. However, the use of Ad5 for gene therapy of IBD may be limited to short-term therapeutic strategies, since commonly used Ad5 constructs are incapable of establishing long-term expression of transgenes and induce immune responses to transduced cells due to the expression of viral genes that are also present in the viral genome. Viral vector species that can provide sustained expression of therapeutic transgenes may be more useful for long-term therapy of IBD.

Adeno-associated viral vectors (AAV) are among the most frequently used viral vector species for *in vivo *gene therapy applications [20-25]. AAV, derived from adeno-associated virus serotype 2 (AAV2), has primarily been used, and extensive effort is being made to modify or engineer its properties, such as the infectivity and tropism. Recent studies are also focused on the development and characterization of AAV constructs that are derived from other serotypes and variants. AAV possesses several attractive characteristics as *in vivo *gene transfer agents: AAV is associated with no known pathogenesis, does not stimulate cell-mediated immune responses, has the ability to transduce a wide range of cell types (i.e., broad tropism), and has the ability to establish long-term expression of transgenes. These multiple attractive properties, particularly its ability to provide sustained expression of transgenes, suggest that AAV may serve as a useful viral vector species for the development of gene therapy strategies for IBD. However, to our knowledge, AAV-based vectors were not previously tested for the transduction of the inflamed colon.

In the present work, we investigated if AAV2 could be used for efficient transduction of the inflamed colon in mice toward its application to gene therapy of IBD. AAV2, when used in free form, showed a limited ability to transduce colonic cell lines *in vitro*. In addition, the transduction efficiency of the inflamed colon in a mouse colitis model was very poor when free AAV2 was administered intracolonically by enema. Thus, we examined if the transduction efficiencies of colonic cell lines and the inflamed colon in mice could be enhanced by the delivery of AAV2 in the form of virus-microbead conjugates bearing lectin as an anchoring agent.

## Results

We initially investigated, by using *in vitro *systems, the infectivity of AAV2 for two colonic cell lines, COLO 205 (human colorectal adenocarcinoma) and MIP-101 (human colorectal carcinoma), along with the human cervical adenocarcinoma cell line HeLa, which is known to be highly permissive to infection by AAV2. We used an AAV2 construct, AAV2.CMV-LacZ, which carries the bacterial *lac*Z (β-galactosidase) gene under the control of the human cytomegalovirus immediate/early promoter. The infectivity of AAV2.CMV-LacZ in free form for these two colonic cell lines was lower by one to two orders of magnitude than that for HeLa cells (data not shown), suggesting that colonic cells may be poorly permissive to infection by AAV2. This result prompted us to investigate if the delivery of AAV2 to target cells in the form of virus-microbead conjugates could enhance the transduction efficiency of colonic cells.

**Figure 1:**
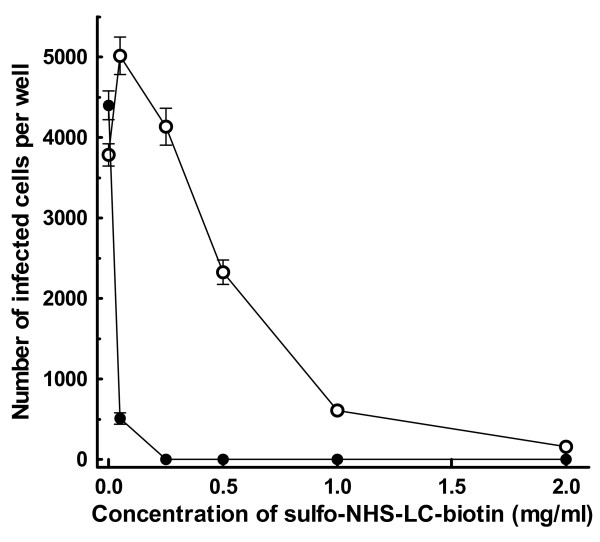
**Infectivity analysis of AAV2 treated with sulfo-NHS-LC-biotin**. AAV2.CMV-LacZ was treated with varying concentrations of sulfo-NHS-LC-biotin at room temperature for 45 min in the dark, followed by the addition of excess glycine to terminate the biotinylation reaction. The resulting biotinylated AAV2 preparations were applied to HeLa cells (5 × 10^6 ^AAV2 particles per well), which had been grown in 24-well plates at 37°C for 24 hr (initial cell number, 5 × 10^4 ^cells per well), and incubated at 37°C for 48 hr. Cells were fixed with glutaraldehyde, stained for β-galactosidase (LacZ) activity using X-gal as the substrate. Then, the number of infected cells, which were stained blue, in each well was counted under a light microscope (-○-). The same analysis was also performed on biotinylated AAV2 preparations, to which excess Neutralite avidin (100 μg per 5 × 10^6 ^AAV2 particles) had been added (-●-). Each datum shown is the average number of infected cells per well with a standard deviation (n = 26).

Preparation of virus-microbead conjugates with AAV2 (AAV2-microbead conjugates) essentially followed the method established for Ad5 [8,26,27] with the optimization of each step. In this method, biotin groups are attached covalently to the surfaces of viral particles by biotinylation using a water-soluble, amino-reactive biotinylation reagent (sulfo-NHS-LC-biotin; Pierce), followed by the removal of non-virion-associated biotinylation reagent. The resulting surface-biotinylated viral particles are attached to microbeads, on which the biotin-binding protein, avidin or streptavidin [28-31], is immobilized covalently in high density (avidin- or streptavidin-coated microbeads, respectively). Optimal conditions for the surface biotinylation of AAV2 particles, where the viral surfaces are biotinylated without disturbing the viral infectivity, were initially determined. AAV2.CMV-LacZ was treated with varying concentrations of sulfo-NHS-LC-biotin at room temperature (~22°C) for 45 min in the dark, followed by the termination of biotinylation reaction. The resulting AAV2 particles were analyzed for their infectivity on HeLa cells that are highly permissive to infection by AAV2 (Fig. 1). The viral infectivity decreased sharply with increasing the concentration of sulfo-NHS-LC-biotin during biotinylation reaction, as seen previously with the biotinylation of Ad5 [26,27,32]. When the concentration of sulfo-NHS-LC-biotin was 0.5 mg/ml or higher, considerable reduction of the viral infectivity was seen (p < 0.01). However, treatment with sulfo-NHS-LC-biotin at concentrations up to 0.25 mg/ml showed a limited effect on the viral infectivity. This suggests that the surfaces of AAV2 particles may be biotinylated by using sulfo-NHS-LC-biotin in this concentration range with minimal effect on the viral infectivity. To test if the viral surfaces were indeed biotinylated, particularly when the concentration of sulfo-NHS-LC-biotin was relatively low, excess avidin was added to AAV2 after biotinylation reaction, and the viral infectivity was then analyzed on HeLa cells. This method allows the assessment of the presence of biotin groups on the viral surfaces, since the viral infectivity is significantly reduced by the binding of avidin to viral-surface biotin groups, as shown previously for biotinylated Ad5 particles [26]. The addition of excess avidin severely reduced the infectivity of AAV2 particles that had been treated with sulfo-NHS-LC-biotin, while it showed little effect on the infectivity of unmodified AAV2.CMV-LacZ (Fig. 1). When AAV2.CMV-LacZ was treated with sulfo-NHS-LC-biotin at concentrations of 0.25 mg/ml and higher, the addition of excess avidin ablated the viral infectivity completely (p < 0.01). When AAV2.CMV-LacZ was treated with 50 μg/ml sulfo-NHS-LC-biotin, the viral infectivity became slightly higher than that of unmodified AAV2.CMV-LacZ (p < 0.01), but the addition of excess avidin reduced the viral infectivity considerably (p < 0.01). This suggests that the treatment of AAV2 with sulfo-NHS-LC-biotin at this concentration can maximally maintain the viral infectivity, while the viral surfaces can indeed be biotinylated. Repeated experiments reveal that the optimal conditions for surface biotinylation of AAV2 particles involve the treatment with sulfo-NHS-LC-biotin at 30 – 70 μg/ml, where the viral infectivity is hardly affected and the majority, if not all, of the viral particles are indeed biotinylated.

**Figure 2:**
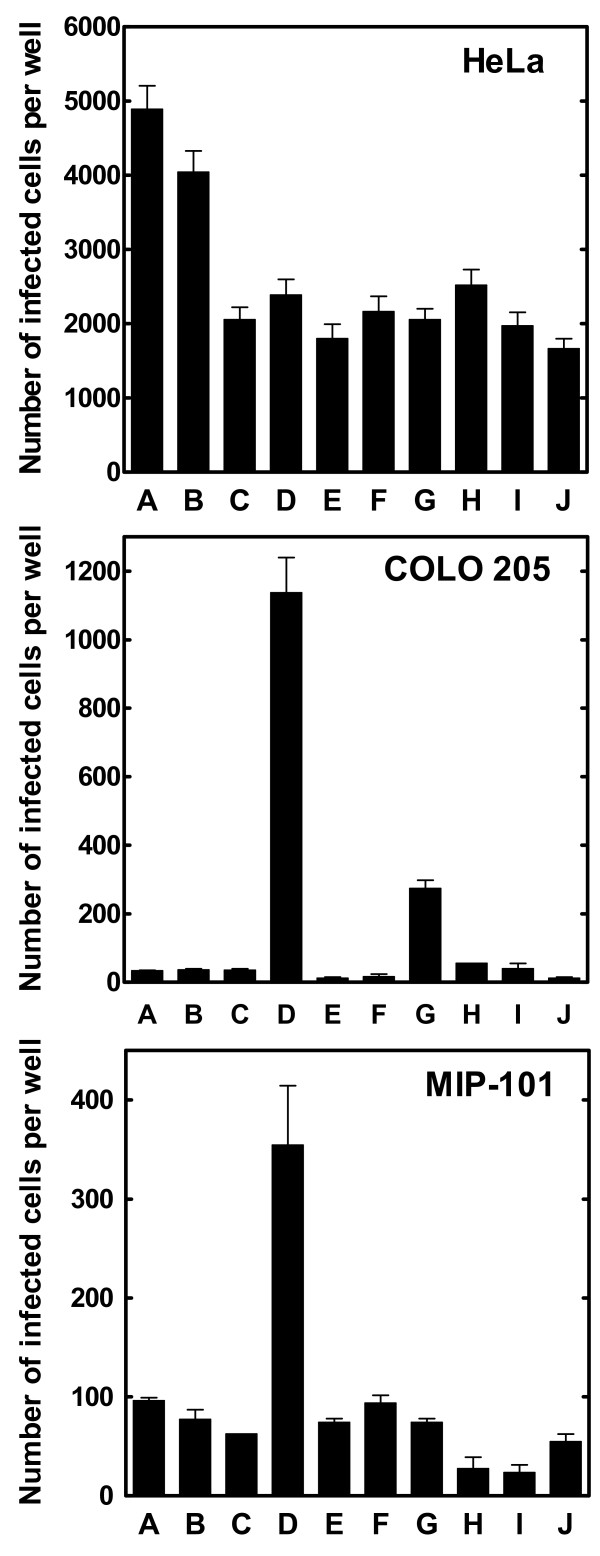
**Effect of the co-attachment of lectins to the microbead surfaces on the infectivity of AAV2-microbead conjugates**. AAV2.CMV-LacZ was biotinylated with sulfo-NHS-LC-biotin at 50 μg/ml, followed by the removal of non-virion-associated biotinylation reagent by dialysis. AAV2-microbead conjugates were prepared by the attachment of biotinylated AAV2 particles to the surfaces of avidin-coated fluorescent microbeads (480 nm in diameter) (9.2 AAV2 particles per microbead). To these AAV2-microbead conjugates, a biotinylated form of each lectin was added in excess (0.2 μg biotinylated lectin per 10^7 ^avidin-coated microbeads), followed by the removal of unbound lectin molecules by centrifugation. The infectivity of these AAV2-microbead conjugates with and without lectin was analyzed on HeLa, COLO 205, and MIP-101 cell lines. Cells were cultured in 24-well plates at 37°C for 24 hr (initial cell number per well: HeLa, 5 × 10^4^; COLO 205, 1 × 10^5^; MIP-101, 7.5 × 10^4^). AAV2-microbead conjugates bearing each lectin, along with free unmodified AAV2.CMV-LacZ, free biotinylated AAV2.CMV-LacZ, and AAV2-microbead conjugates without lectin, were applied to target cells (a total of 1 × 10^7 ^AAV2 particles per well) and incubated at 37°C for 48 hr. Cells were fixed with glutaraldehyde and stained for β-galactosidase activity using X-gal as the substrate. Then, the number of infected cells in each well was counted under a light microscope. Each datum shown is the average number of infected cells per well with a standard deviation (n = 16). A, free, unmodified AAV2.CMV-LacZ; B, free, biotinylated AAV2.CMV-LacZ; C, AAV2-microbead conjugates without lectin; D – J, AAV2-microbead conjugates bearing lectin (D, Con A; E, horse gram agglutinin; F, peanut agglutinin; G, castor bean agglutinin I; H, soybean agglutinin; I, furze gorse agglutinin I; and J, wheat germ agglutinin).

Preparation of AAV2-microbead conjugates was carried out by using AAV2.CMV-LacZ that had been treated with 50 μg/ml sulfo-NHS-LC-biotin as above. After the termination of biotinylation reaction, biotinylated AAV2 particles were dialyzed to remove non-virion-associated biotinylation reagent and mixed with avidin-coated fluorescent microbeads, in which a rhodamine derivative is encapsulated (diameter, 480 nm; specific gravity, 1.06 g/cm^3^) (Spherotech). Infectivity analysis of the supernatant after the centrifugation of the mixtures revealed that greater than 95% of the AAV2 particles used were bound to avidin-coated microbeads, indicating that the binding of biotinylated AAV2 particles to avidin-coated microbeads was highly efficient. The resulting AAV2-microbead conjugates (9.2 AAV2 particles per microbead) were analyzed for their infectivity on HeLa, COLO 205, and MIP-101 cells. The infectivity of these AAV2-microbead conjugates was lower than or equivalent to free, unmodified AAV2.CMV-LacZ (HeLa, p < 0.01; COLO 205 and MIP-101, p > 0.05) (Fig. 2). This suggests that the ability of these conjugates to associate with target cells may be similar to that of free AAV2 particles, since the microbeads, used as carriers of AAV2 particles, have physiological specific gravity. This notion was supported by fluorescence microscopic analysis of target cells, in which only limited amounts of cell-associated fluorescence, derived from the avidin-coated microbeads used, were seen after AAV2-microbead conjugates were applied and incubated for 24 hr (data not shown).

We previously showed with Ad5-microbead conjugates [8,27] that the co-attachment of a lectin, Con A, to the microbead surface allowed the conjugates to associate stably with target cells due to the ability of Con A to bind to commonly occurring α-D-mannosyl moieties in cell-surface carbohydrate chains, resulting in efficient transduction of the target cells. We assessed if the ability of AAV2-microbead conjugates to associate with target cells and subsequently transduce them could also be enhanced by the co-attachment of lectins on the microbead surfaces. AAV2-microbead conjugates were prepared as above (9.2 AAV2 particles per microbead), and then biotinylated forms of several lectins with different carbohydrate specificities (see the Methods section) were co-attached to the microbead surfaces. These conjugates were analyzed for their infectivity on HeLa, COLO 205, and MIP-101 cells (Fig. 2). For HeLa cells, which are highly permissive to infection by free AAV2, the co-attachment of lectins to the microbead surface showed little effect on the infectivity of AAV2-microbead conjugates (p > 0.05 for all of the lectins used). In contrast, the infectivity of AAV2-microbead conjugates on COLO 205 and MIP-101 cells was enhanced considerably when certain lectins were co-attached to the microbead surfaces. In particular, the co-attachment of Con A showed dramatic enhancement of the infectivity of AAV2-microbead conjugates for these colonic cell lines (p < 0.01 for both COLO 205 and MIP-101). When COLO 205 cells were used as targets, the infectivity of AAV2-microbead conjugates bearing Con A became approximately 30-fold higher than those of free, unmodified AAV2 or AAV2-microbead conjugates without lectin (p < 0.01). Similarly, the co-attachment of Con A enhanced the infectivity of AAV2-microbead conjugates for MIP-101 cells by approximately 4-fold, as compared to free, unmodified AAV2 or AAV2-microbead conjugates without lectin (p < 0.01). No appreciable effect on the morphology and viability of target cells was seen for any of these cell lines when Con A was co-attached to the microbead surfaces. Other lectins tested showed little effect on the infectivity of AAV2-microbead conjugates on COLO 205 and MIP-101 cells, except for castor bean agglutinin I that enhanced the infectivity of the conjugates for COLO 205 cells by approximately 8-fold (p < 0.01). The considerable infectivity enhancement of AAV2-microbead conjugates by the co-attachment of Con A for these poorly permissive colonic cell lines may be derived from the fact that Con A binds to α-D-mannosyl moieties that are commonly and abundantly present in cell-surface carbohydrate chains, allowing the conjugates to associate with target cells with much higher efficiencies.

**Figure 3:**
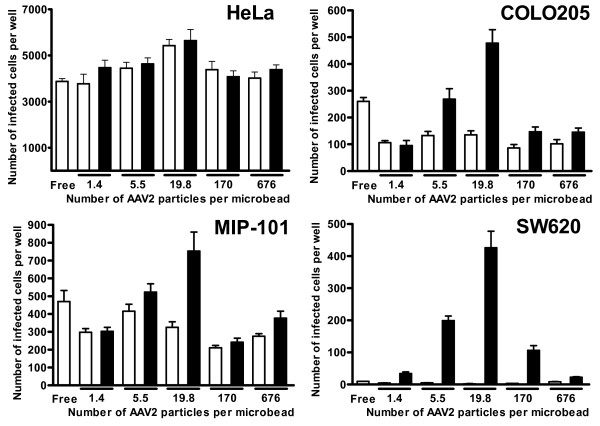
**Effect of the number of AAV2 particles per microbead on the infectivity of AAV2-microbead conjugates with and without Con A**. AAV2-microbead conjugates were prepared as in Fig. 2 at varying numbers of AAV2 particles per microbead (0 – 676 AAV2 particles per microbead). These conjugates with and without the co-attachment of Con A were analyzed for their infectivity on HeLa, COLO 205, MIP-101, and SW620 cell lines. Cells were cultured in 24-well plates at 37°C for 24 hr (initial cell number per well: HeLa, 5 × 10^4^; COLO 205, 1 × 10^5^; MIP-101, 7.5 × 10^4^; SW620, 1 × 10^5^). AAV2-microbead conjugates with and without Con A, along with free, unmodified AAV2.CMV-LacZ, were applied to target cells (1 × 10^7 ^AAV2 particles per well for HeLa, and 1 × 10^8 ^AAV2 particles per well for COLO 205, MIP-101, and SW620) and incubated at 37°C for 48 hr. Cells were fixed with glutaraldehyde and stained for β-galactosidase activity using X-gal as the substrate. Then, the number of infected cells in each well was counted under a light microscope. Open bars, without Con A; solid bars, with Con A. Each datum shown is the average number of infected cells per well with a standard deviation (n = 16).

The effect of the number of AAV2 particles per microbead on the infectivity of AAV2-microbead conjugates bearing Con A was analyzed for the three cell lines used above, along with a human colorectal adenocarcinoma cell line, SW620, which was previously found to have an even lower permissivity to infection by free AAV2 than COLO 205 and MIP-101 cells. AAV2-microbead conjugates were prepared with varying numbers of AAV2 particles per microbead (0 – 676 AAV2 particles per microbead). The infectivity of these conjugates with and without the co-attachment of Con A, along with free, unmodified AAV2.CMV-LacZ as a control, was analyzed (Fig. 3). In this analysis, the amount of total AAV2 particles per well was kept constant for each cell line. This means that the total number of AAV2-microbead conjugates per well decreased with increasing the number of AAV2 particles per microbead. For HeLa cells that are highly permissive to infection by free AAV2, the number of AAV2 particles per microbead had little effect on the infectivity of AAV2-microbead conjugates, which was similar to that of free AAV2. The co-attachment of Con A to the microbead surfaces also showed little effect on the infectivity of the conjugates (p > 0.05 for all of the numbers of AAV2 particles per microbead tested), suggesting that the infection process was initiated primarily by direct binding of AAV2 particles on the microbead surfaces to cell-surface heparan sulfate, not by the binding of Con A on the microbead surfaces to cell-surface carbohydrate chains. In contrast, the number of AAV2 particles per microbead showed a dramatic effect on the infectivity of the conjugates for the three colonic cell lines, particularly when Con A was co-attached to the microbead surfaces. For COLO 205 and MIP-101 cells, the number of AAV2 particles per microbead had a limited effect on the infectivity when AAV2-microbead conjugates were used without the co-attachment of Con A. However, when Con A was co-attached to the microbead surfaces, the infectivity of the conjugates became higher than those without Con A, and the highest infectivity was seen when the number of AAV2 particles per microbead was 19.8 for both COLO 205 and MIP-101 cell lines. Similarly, both free AAV2 and AAV2-microbead conjugates without Con A had extremely low infectivity for SW620 cells, but the co-attachment of Con A to the microbead surfaces substantially enhanced the infectivity of AAV2-microbead conjugates (p < 0.01 for all of the numbers of AAV2 particles per microbead tested, except at 676 AAV2 particles per microbead), particularly when the number of AAV2 particles per microbead was 19.8. Repeated experiments reveal that the infectivity of AAV2-microbead conjugates bearing Con A for these poorly permissive colonic cell lines became maximal when the number of AAV2 particles per microbead was in a range between 10 and 60.

**Figure 4:**
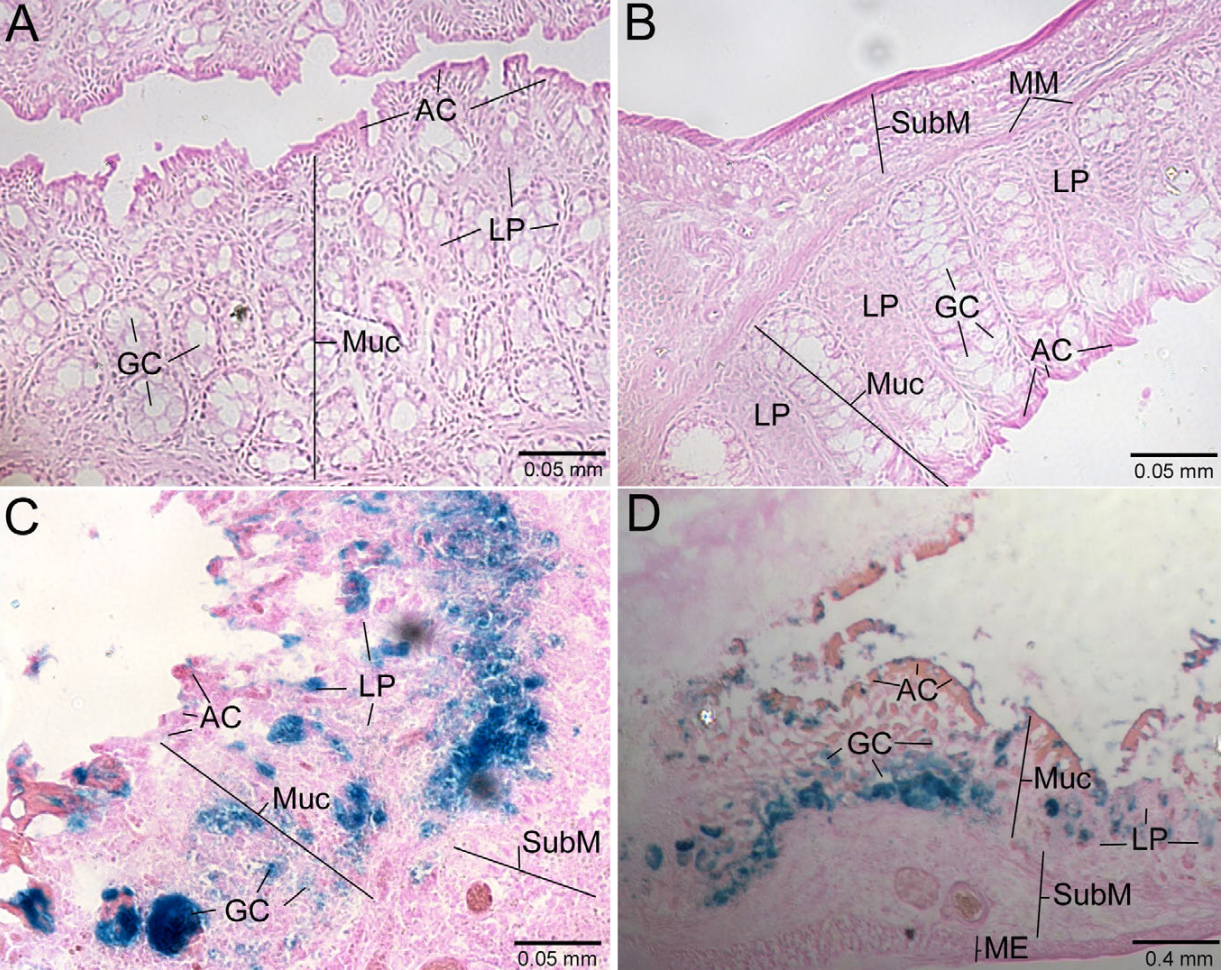
**Transduction of the inflamed colon in mice by AAV2-microbead conjugates bearing Con A upon intracolonical administration**. AAV2-microbead conjugates with and without Con A were prepared at 32 AAV2 particles per microbead, as described in Figs. 3 and 4. These conjugates, along with free, unmodified AAV2.CMV-LacZ, were administered intracolonically into mice with TNBS-induced colitis by enema (a total of 1 × 10^10 ^AAV2 particles in 100 μl PBST per mouse). At 48-hr post-administration, mice were euthanized, and cryosections of their colons were prepared. These colon sections were stained for β-galactosidase activity using X-gal as the substrate, followed by counter-staining with eosin/phloxine B. Stained colon sections were examined under a light microscope. A, free, unmodified AAV2.CMV-LacZ; B, AAV2-microbead conjugates without Con A; C and D, AAV2-microbead conjugates bearing Con A. AC, absorptive cell; GC, goblet cell; LP, lamina propria; ME, muscularis externa; MM, muscularis mucosa; Muc, mucosal layer; SubM, sub-mucosal layer. Representative images are shown.

The results of *in vitro *experiments described above suggest that AAV2-microbead conjugates bearing Con A may offer enhanced transduction of the colon over free AAV2 and AAV2-microbead conjugates without lectin when administrated intracolonically by enema. To test this, we used a mouse acute colitis model, in which colonic inflammation was induced chemically by intracolonical administration of 2,4,6-trinitrobenzenesulfonic acid (TNBS) [8,33]. AAV2-microbead conjugates were prepared using AAV2.CMV-LacZ at 32 AAV2 particles per microbead. These conjugates with the co-attachment of Con A were administered intracolonically into mice with TNBS-induced colitis by enema [a total of 1 × 10^10 ^AAV2 particles per mouse]. As controls, free, unmodified AAV2.CMV-LacZ and AAV2-microbead conjugates without Con A were administered in the same manner. At 48-hr post-administration, mice were euthanized, and their colons were collected, fixed, and sectioned. These colon sections were stained for *lac*Z expression using 5-bromo-4-chloro-3-indoyl-β-D-galactopyranoside (X-gal) as the substrate (Fig. 4). When free AAV2.CMV-LacZ was administered intracolonically, no appreciable expression of the *lac*Z gene was seen (Fig. 4A), indicating the low permissivity of colonic tissue to infection by free AAV2. The transduction efficiency of the inflamed colon remained very low when AAV2.CMV-LacZ was used in the form of AAV2-microbead conjugates without Con A (Fig. 4B). In contrast, intracolonical administration of AAV2.CMV-LacZ in the form of AAV2-microbead conjugates bearing Con A resulted in very efficient transduction of the colonic tissue (Figs. 4C and 4D). Transduction was seen primarily in the mucosal layer, to which administered AAV2-microbead conjugates should have easy access due to its destruction caused by colonic inflammation. Absorptive and goblet cells in the colon epithelium, along with lamina propria in crypts, were primarily transduced. In addition, the muscularis mucosa and the sub-mucosal layer may also be transduced, suggesting that AAV2-microbead conjugates not only infected the surfaces of colonic epithelia but also migrated into and subsequently transduced colonic tissue. Since colonic epithelial cells actively turn over and are rapidly shed, the transduction of these cells alone should provide only short-term production of therapeutic proteins in the inflamed colon, offering limited effectiveness for the therapy of IBD. In contrast, the transduction of other cell types in the muscularis mucosa and the sub-mucosal layer should offer sustained expression of transgenes, which should be useful for the development of long-term therapeutic strategies for IBD.

**Figure 5:**
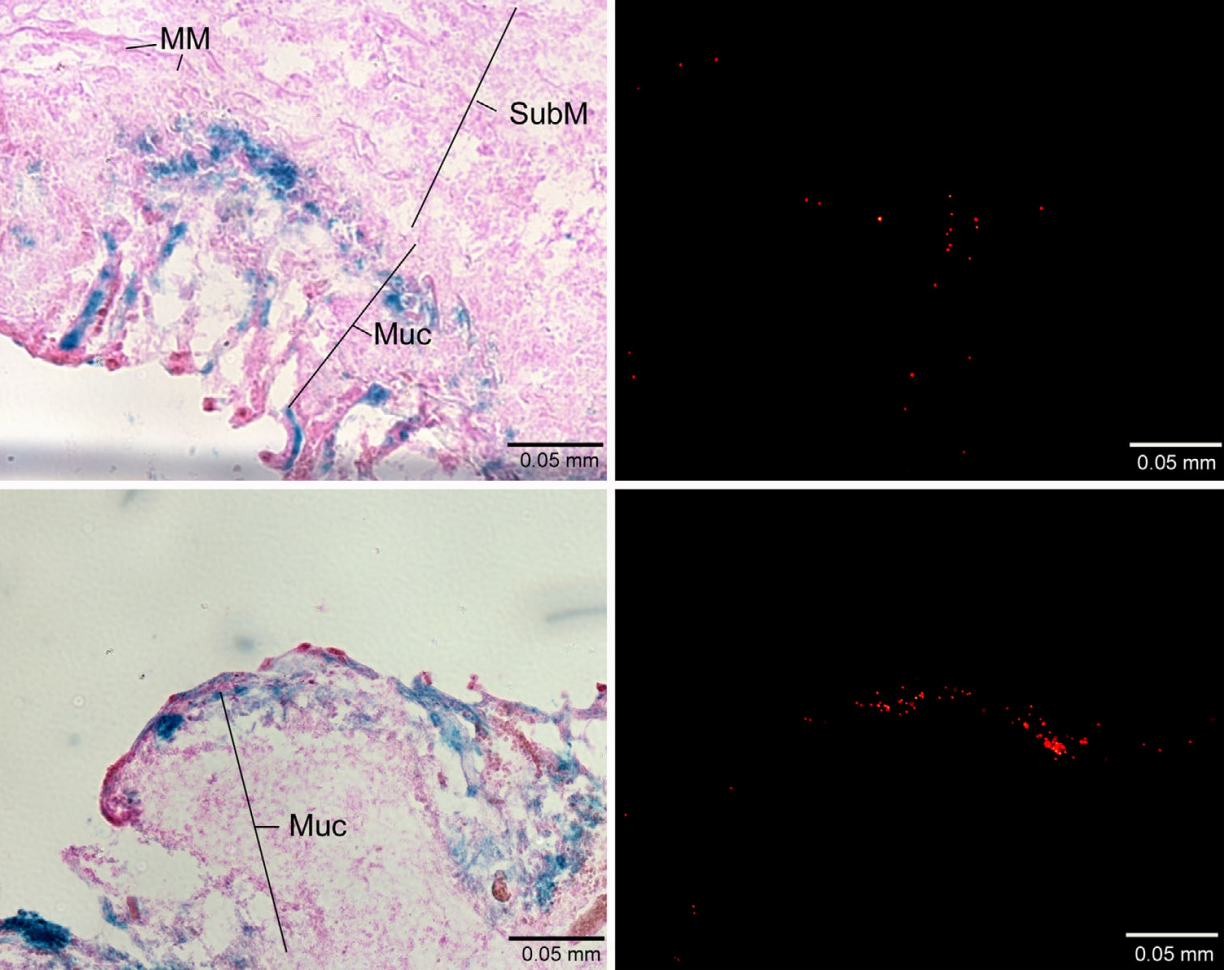
**Spatial relationship between transduction sites and the location of tissue-associated microbeads in the inflamed colon upon intracolonical administration of AAV2-microbead conjugates bearing Con A**. Colon sections, prepared from mice that received AAV2-microbead conjugates with and without the co-attachment of Con A, were stained for β-galactosidase activity using X-gal as the substrate. Stained sections (without counter-staining) were examined under a fluorescence microscope to detect microbead-derived red fluorescence (right images). Then, colon sections were counter-stained with eosin/phloxine B and subjected to light microscopic analysis for transduction sites (left images), in which images at the same location as fluorescence detection of tissue-associated microbeads above were captured. Colon sections, derived from mice that received AAV2-microbead conjugates without Con A, showed no appreciable transduction or tissue-associated microbeads (data not shown). The two pairs of images shown are derived from colon sections of mice that received AAV2-microbead conjugates bearing Con A. MM, muscularis mucosa; Muc, mucosal layer; SubM, sub-mucosal layer. Representative images are shown.

We also investigated the spatial relationship between transduction sites and the location of tissue-associated microbeads in colon sections from mice, which received AAV2-microbead conjugates with and without the co-attachment of Con A (Fig. 5). Colon sections were initially stained for *lac*Z expression using X-gal as the substrate, and stained sections were examined under a fluorescence microscope to detect microbead-derived red fluorescence. Few microbeads were detected in colonic tissue, which received AAV2-microbead conjugates without Con A (data not shown). This suggests that these conjugates have a limited ability to associate with colonic tissue upon intracolonical administration. In contrast, many tissue-associated microbeads were seen in colon sections when AAV2 was used in the form of AAV2-microbead conjugates bearing Con A (Fig. 5), suggesting that the co-attachment of Con A allowed the conjugates to associate stably with colonic tissue upon intracolonical administration. The same colon sections were then counter-stained with eosin/phloxine B and examined under a light microscope to detect the transduction by AAV2. Efficient transduction was seen at locations where many tissue-associated microbeads were present. This reveals that the co-attachment of Con A makes AAV2-microbead conjugates capable of associating stably with colonic tissue, allowing the attached AAV2 particles to mediate efficient transduction of colonic cells.

## Discussion

In the present study, we have demonstrated that AAV2-microbead conjugates bearing Con A can serve as efficient gene transfer agents both for colonic cell lines *in vitro *and for the inflamed colon in a mouse colitis model. Con A was found to be an effective anchoring agent for AAV2-microbead conjugates, providing efficient transduction of target cells and tissues both *in vitro *and *in vivo*. The infectivity enhancement by the co-attachment of Con A is likely derived from the ability of Con A on the microbead surfaces to allow the conjugates to associate stably with target cells or tissues due to its ability to bind to commonly occurring α-D-mannosyl moieties in cell-surface carbohydrate chains. The ability to associate stably with target tissue may be particularly important for the transduction of the colorectal system, where the dynamic fluidic properties inhibit stable association of viral particles with colonic tissues. We also analyzed the transduction of the normal colons (i.e., without colonic inflammation) in mice by AAV2 upon intracolonical administration. Under the conditions used for the transduction of the colons with TNBS-induced colitis (Fig. 4), no appreciable transduction of the normal colons was seen when AAV2 was used either in free form or in the form of AAV2-microbead conjugates with or without the co-attachment of Con A (data not shown). These results suggest that the exposure of colonic tissue by the destruction of the mucous coat on the colonic epithelium upon the development of TNBS-induced colitis is critical for AAV-microbead conjugates bearing Con A to associate stably to the colonic tissue and subsequently transduce colonic cells.

A different strategy for the construction of AAV2-microbead conjugates was previously reported [34]. In this strategy, microbeads are coated with the primary receptor of AAV2, heparan sulfate, to which unmodified AAV2 particles are attached. We tested this strategy *in vitro *by using avidin-coated microbeads with physiological specific gravity, used in this study, with unmodified AAV2.CMV-LacZ. No appreciable enhancement of the infectivity was seen with these AAV2-microbead conjugates, as compared to free AAV2.CMV-LacZ, when analyzed using cultured colonic cell lines (data not shown). This may be derived from the physiological specific gravity of such conjugates, with which their ability to associate with target cells may be similar to that of free AAV2, as seen with AAV2-microbead conjugates without lectin in this study. The presence of large amounts of heparan sulfate on the microbead surfaces may also inhibit efficient binding of attached AAV2 particles to cell-surface heparan sulfate. In addition, this strategy does not allow the co-attachment of other materials on the microbead surfaces, to which AAV2 particles have been conjugated. Thus, the use of such conjugates for the transduction of the inflamed colon may be limited, since their association with colonic tissue upon intracolonical administration should be inefficient without the co-attachment of an appropriate anchoring agent, such as Con A. In contrast, a key strength of our strategy is that almost any materials can readily be co-attached to the microbead surfaces, to which AAV2 particles (or other viral particles used) have been conjugated. This should allow the properties and functionality of AAV2-microbead conjugates to be controlled or engineered in a systematic manner. In addition, our strategy should be applicable to AAV-based vectors that are derived from not only AAV2 but also other serotypes and variants. This should make our strategy applicable to the transduction of a wide range of cell types and tissue targets.

## Conclusion

This study has demonstrated that the delivery of AAV2 in the form of AAV2-microbead conjugates bearing the lectin Con A provides considerable enhancements of the transduction efficiency both for poorly permissive colonic cell lines *in vitro *and for the inflamed colon in mice upon intracolonical administration by enema. In particular, the co-attachment of Con A to the microbead surfaces as an anchoring agent was found essential for efficient transduction of colonic cells by AAV2-microbead conjugates both *in vitro *and *in vivo*. With this efficient AAV2-mediated transduction system for the inflamed colon, it should now be possible to investigate, systematically, the efficacy of potential gene therapy strategies for IBD by using the genes for a variety of different protein therapeutics. Such protein therapeutics should include the anti-inflammatory cytokine IL-10, antibodies against the pro-inflammatory cytokine TNF-α, and the extracellular domain of the TNF-α receptor, each of which has shown potential effectiveness for the therapy of IBD.

## Methods

### AAV2 construct

An AAV2 construct, AAV2.CMV-LacZ, was obtained from the Vector Core, Harvard Gene Therapy Initiative, Dana-Farber/Harvard Cancer Center, Boston, MA. AAV2.CMV-LacZ, derived from adeno-associated virus serotype 2, carries the bacterial *lac*Z (β-galactosidase) gene with a nuclear localization signal under the control of the human cytomegalovirus immediate/early promoter with the polyadenylation signal of the bovine growth hormone gene. Prior to use, AAV2.CMV-LacZ was purified twice by affinity chromatography using heparin as the ligand [35] (heparin-agarose type I; Sigma).

### Cell lines

The following four human cell lines were used as targets for *in vitro *experiments: HeLa (cervical adenocarcinoma), COLO 205 (colorectal adenocarcinoma), MIP-101 (colorectal carcinoma), and SW620 (colorectal adenocarcinoma). HeLa, COLO 205, and SW620 were obtained from the American Type Culture Collection (Manassas, VA), and MIP-101 was a generous gift from Peter Thomas, Boston University School of Medicine, Boston, MA. HeLa and SW620 cells were maintained in Dulbecco's modified Eagle's medium (BioWhittaker), supplemented with 10% fetal bovine serum (BioWhittaker). COLO 205 and MIP-101 cells were maintained in RPMI 1640 medium (BioWhittaker), supplemented with 10% fetal bovine serum, 4.5 mg/ml glucose, 1.5 mg/ml sodium bicarbonate, and 10 mM 4-(2-hydroxyethyl)-1-piperazineethanesulfonic acid.

### Biotinylation of AAV2 particles

A water-soluble, amino-reactive biotinylation reagent, sulfo-NHS-LC-biotin (Pierce) [8,26,27,32], was used to biotinylate AAV2 particles. Sulfo-NHS-LC-biotin was freshly dissolved in Dulbecco's phosphate-buffered saline (PBS) containing 0.05% Tween 20 (PBST) and added to AAV2.CMV-LacZ in PBST (1 × 10^10 ^AAV2 particles/ml) at varying final concentrations (0 – 2 mg/ml). The mixtures were incubated at room temperature (~22°C) for 45 min in the dark, and the biotinylation reaction was terminated by the addition of glycine in PBS to a final concentration of 10 mM. The resulting biotinylated AAV2 particles were analyzed for their infectivity by using HeLa cells as the targets. HeLa cells were grown in 24-well plates (5 × 10^4 ^cells per well) at 37°C for 24 hr. Then, each biotinylated AAV2.CMV-LacZ preparation was applied to target cells (5 × 10^6 ^AAV2 particles per well containing 500 μl of culture media) and incubated at 37°C for 48 hr. Cells were fixed with 0.5% glutaraldehyde and stained for β-galactosidase (LacZ) activity using X-gal as the substrate. The numbers of infected, *lac*Z-expressing cells were counted under a light microscope. The same analysis was also performed by using biotinylated AAV2.CMV-LacZ preparations, to which excess amounts of Neutralite avidin (Southern Biotechnology Associates) (100 μg per 5 × 10^6 ^AAV2 particles) had been added, to assess the biotinylation of the viral surfaces.

### Preparation of AAV2-microbead conjugates with and without the co-attachment of lectin

Preparation of AAV2-microbead conjugates was carried out by using AAV2.CMV-LacZ and avidin-coated fluorescent polystyrene microbeads (diameter, 480 nm; specific gravity, 1.06 g/cm^3^) (Spherotech), in which a rhodamine derivative (red fluorescence) is encapsulated. AAV2.CMV-LacZ was biotinylated with sulfo-NHS-LC-biotin at 50 μg/ml as above. After the termination of biotinylation reaction by the addition of glycine, biotinylated AAV2 particles were dialyzed against PBST to remove non-virion-associated biotinylation reagent. Dialyzed AAV2 particles were mixed with avidin-coated microbeads at appropriate ratios. The mixtures were incubated at room temperature for 30 min with occasional mixing, followed by the removal of unbound AAV2 particles by centrifugation. The supernatant fraction, which contained unbound AAV2 particles, was analyzed for its infectivity on HeLa cells to determine the number of bound AAV2 particles per microbead.

The following lectins in biotinylated form were obtained from Vector Laboratories [the carbohydrate structure(s), to which each lectin binds, is indicated in bracket]: Con A from Jack bean (*Canavalia ensiformis*) seeds [mannose]; agglutinin from horse gram (*Dolichos biflorus*) seeds [*N*-acetylgalactosamine]; agglutinin from peanuts (*Arachis hypogaea*) [galactosyl (β-1,3)*N*-acetylgalactosamine]; agglutinin I from castor bean (*Ricinus communis*) seeds [galactose and *N*-acetylglucosamine]; agglutinin from soybean (*Glycine max*) seeds [*N*-acetylgalactosamine and galactose]; agglutinin I from furze gorse (*Ulex europaeus*) seeds [fucose]; and agglutinin from wheat germ (*Triticum vulgaris*) [*N*-acetylglucosamine]. The co-attachment of a lectin to the microbead surface was carried out by the addition of an excess amount of a biotinylated form of the lectin to AAV2-microbead conjugates (0.2 μg biotinylated lectin per 10^7 ^avidin-coated microbeads), followed by the removal of unbound lectin molecules by centrifugation.

### Infectivity analysis of AAV2-microbead conjugates with and without lectin

The infectivity of AAV2-microbead conjugates with and without the co-attachment of lectin, prepared above, was analyzed by using HeLa, COLO 205, MIP-101, and SW620 cell lines as targets. Cells were cultured in 24-well plates at 37°C for 24 hr. AAV2-microbead conjugates with and without lectins, along with free, unmodified AAV2.CMV-LacZ and free, biotinylated AAV2.CMV-LacZ as controls, were applied to each well. The actual numbers of target cells and AAV2 particles per well for each cell line are given in the legends to Figs. 2 and 3. Cells were incubated at 37°C for 48 hr, fixed with glutaraldehyde, and stained for β-galactosidase activity using X-gal as the substrate. Then, the numbers of infected cells were counted under a light microscope.

### *In vivo* transduction of the inflamed colon in mice by AAV2-microbead conjugates

All animal procedures were carried out in accordance with NIH guidelines following approval by the Harvard Medical Area Standing Committee on Animals. A mouse acute colitis model was prepared by intracolonical administration of TNBS (Sigma) (0.75 mg in 100 μl of 50% ethanol per mouse) into Balb/c mice (6 – 8 weeks old; Taconic) by enema, as described previously [8]. These mice with TNBS-induced colitis were subjected to experimentation at 48-hr post-administration of TNBS, at which time severe destruction of the mucosal layer was seen in the colon and the mice lost their weight by 10 – 15% during the 48-hr period. AAV2-microbead conjugates bearing Con A were prepared by using AAV2.CMV-LacZ at 32 AAV2 particles per microbead, as described above. These conjugates were administered intracolonically into mice with TNBS-induced colitis by enema (a total of 1 × 10^10 ^AAV2 particles in 100 μl PBST per mouse). As controls, free, unmodified AAV2.CMV-LacZ and AAV2-microbead conjugates without Con A were administered into mice with TNBS-induced colitis in the same manner. At 48-hr post-administration, mice were euthanized, and their colons were collected. These colons samples were frozen in tissue freezing media (Tissue-Tek O.C.T. compound, Miles), followed by the preparation of cryosections (thickness, 5 – 7 μm). Colon sections were stained for β-galactosidase activity using X-gal as the substrate, with counter-staining with eosin/phloxine B. Stained colon sections were examined under a light microscope (Axioscop 2, Carl Zeiss).

When the spatial relationship between transductions sites and the location of tissue-associated microbeads was analyzed, colon sections were stained for β-galactosidase activity using X-gal as the substrate. Stained colon sections (without counter-staining) were initially examined under a fluorescence microscope (Axioscop 2) to detect tissue-associated microbeads (red fluorescence). Then, the colon sections were counter-stained with eosin/phloxine B and examined under a light microscope (Axioscop 2) for the detection of transduction sites, where images at the same location as fluorescence detection of tissue-associated microbeads above were captured.

## Authors' contributions

SJF carried out *in vitro *experiments for the characterization of AAV2 and its microbead conjugates. AJ performed animal experiments with a mouse colitis model, including *in vitro *analysis of tissue samples. TS conceived of the study and directed the research group. AJ and TS drafted the manuscript. All authors read and approved the final manuscript.
